# Phytochemical composition and health properties of Sembung plant (*Blumea balsamifera*): A review

**DOI:** 10.14202/vetworld.2021.1185-1196

**Published:** 2021-05-17

**Authors:** I. Gede Widhiantara, I. Made Jawi

**Affiliations:** 1 Medical Science Study Program, Faculty of Medicine, Udayana University, Jalan P.B. Sudirman, Denpasar City, Bali Province 80234, Indonesia; 2 Study Program of Biology, Faculty of Health, Science, and Technology, Dhyana Pura University, Jalan Raya Padang Luwih, Dalung, North Kuta, Badung, Bali Province 80361, Indonesia; 3 Department of Pharmacology, Faculty of Medicine, Udayana University, Jalan P.B. Sudirman, Denpasar City, Bali Province 80234, Indonesia

**Keywords:** Asteraceae, *Blumea balsamifera*, future medicine, medicinal uses, phytochemical constituents

## Abstract

Indonesia’s mindset has been focusing on the use of natural medicines, food, and healing practices widely recognized by the nation’s culture. Traditional medicines and herbs used in traditional medicine can often lead to the discovery of drugs against certain diseases. The aim of this review was to study evidence-based data on the importance of Sembung plant, *Blumea balsamifera*, as a potential traditional medicine. The distribution, ethnopharmacology, secondary metabolites, and bioactivity against several diseases are focused in this review. Information and research related to Sembung plant were searched using the terms *“B. balsamifera*,” “phytochemicals,” and “pharmacological activity” on ResearchGate, Google Scholar, Science Direct, PubMed, and scientific information-based databases up to 2020. Several ethnomedical articles recommend *B. balsamifera* for the treatment of sinusitis, colic pain, cough, kidney stones, flu, or as a diuretic. This knowledge has already been applied in several countries in Southeast Asia. *B. balsamifera* has been reported to contain several phytochemicals both volatile (terpenoids, fatty acids, phenols, alcohol, aldehydes, ethers, ketones, pyridines, furans, and alkanes) and non-volatile (flavonoids, flavanones, and chalcones). Extracts and phytochemicals of *B. balsamifera* contain several biological capacities such as antioxidant, antimicrobial, antifungal, anti-inflammatory, hypolipidemic, anti-infertility, hepatoprotective activity, antidiabetic, gastroprotective, antitumor, anticancer, and immunomodulatory agent against Coronavirus disease-19 infection. This review provides essential data for the potential application of *B. balsamifera* as a nutraceutical or in future medicinal preparations.

## Introduction

Indonesia has enormous potential for the development of herbal medicines and traditional medicinal preparations that have been already traditionally used to treat various diseases [1]. Apart from Indonesia, the use of traditional medicines has also been developed in other countries of the Southeast Asian Association (ASEAN) in recent years [2]. The use of these traditional plants has been targeting several diseases, from a common cold to cancer [3]. Contemporary research has validated several parts of plants from roots, stems, and leaves in potential medicinal preparations by screening for active ingredients such as plant metabolites [4-6].

*Blumea balsamifera* (L) DC. (Asteraceae) or known as local Sembung (in Indonesia) has been widely used as a traditional medicinal preparation for thousands of years. Several countries in Southeast Asia, such as China, Malaysia, Thailand, Vietnam, and the Philippines, have also used the Sembung plant as a traditional medicine [7]. Species from the genus Blumea are distributed across tropical Asia, Africa, and Oceania [8], with the highest level of diversity in tropical Asia [9,10], including Indonesia. To date, as many as 49 species of Blumea are distributed worldwide, with 27 found in Southeast Asia [11]. In the last decade, researchers have focused their investigations on species from the genus Blumea that are used in Ayurvedic (Indian) medicinal ingredients and traditional drinks (Loloh) by Balinese people in Indonesia [12,13].

Therefore, this review provides evidence-based information on the potential biological activity of *B. balsamifera*, not only toward a central role as a nutraceutical in traditional medicine but also as an interesting plant to undergo further phytochemical and pharmacological studies.

## Traditional Use in Several Countries

**Figure-1:**
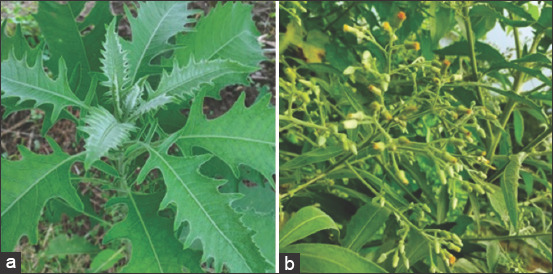
Sembung plants (*Blumea balsamifera*). (a) Leaves and (b) Flowers.

*B. balsamifera* is a wild terrestrial plant that can grow to an altitude of 2200-3500 m asl in humid to dry areas (Figure-1) [14]. *B. balsamifera* has many names in several countries such as Ainaxiang and Dafeng “ai” in China, and it is commonly used as an incense because of its high content in essential oil [15]. In Thailand, dried leaves of the Sembung (Naat) plant can be used as a cigarette to relieve sinusitis, colic pain, and cough and can be combined with other plants as a bath ingredient for women after giving birth [16]. In addition, Thai population also believes that Sembung “drives away spirits.” In the Philippines, people are more familiar with the name Sambong, used as a traditional medicine for patients with kidney stones, common cold or as a diuretic [17]. Other Asian countries such as Malaysia and India also use Sembung as an Ayurvedic medicine [18,19].

The use of the Sembung plant in traditional medicine in Indonesia is also known to be very diverse. Within Indonesia, this plant is known by different local names: Sembung utan (Sundanese), Sembung gantung, kuwuk, mingsa, langu (Java), Kamadhin (Madura), Sembung (Bali), Capo (Sumatra), and Afoat, Ampampau, and Madikapu (Eastern Indonesia) [20]. The Sembung plant can be used as a traditional drink by Balinese people called “Loloh Sembung” [21]. Loloh Sembung is made by boiling and brewing fresh and dried leaves [13].

## Progress on Phytochemical Studies Using *B. balsamifera*

Studies show that more than 100 phytochemical constituents of *B. balsamifera* can be both volatile and non-volatile. The volatile constituent compounds consist of terpenoids, fatty acids, phenols, alcohols, aldehydes, ether, ketones, pyridine, furans, and alkanes. Flavonoids, flavanones, and chalcones are non-volatile constituents [7]. The diversity of the constituents found in *B. balsamifera* has potential medicinal benefits, as shown in Table-1 [22-54].

**Table-1:** The diversity of constituents, molecular formulas, and bioactivity.

Volatile constituents			

Class	Molecular formula*	Bioactivity and medical benefit	Reference
Terpenoids	C5H8	Inhibition of NO production induced by LPS in RAW264.7 macrophages	[24]
Fatty acid	CH_3_(CH_2_)_ n_COOH (with n variation)	Fatty acids, especially unsaturated fatty acids with n-3 and n-6 have good bioactive and nutritional compounds and play an important role in lipid homeostasis and cardiovascular disease prevention, prevention of chronic disease, anti-inflammatory	[25-28]
Phenol	C_6_H_6_O or C_6_H_5_OH	Has antibacterial activity against *S. aureus* and high antioxidant activity, procoagulants	[23,29]
Alcohol	CH_3_CH_2_OH or C_2_H_6_O	Has good antibacterial activity	[30]
Aldehydes	RCHO	Anticancer and anti- inflammatory	[31,32]
Ether	C_4_H_10_O or (C_2_H_5_)_ 2_O or CH_3_CH_2_OCH_2_CH_3_	As a natural anesthetic agent	[33]
Ketones	CH_3_COCH_3_	Anticancer, antimicrobial, and antioxidant activity	[34-36]
Pyridine	C_5_H_5_N	Anticancer and phosphodiesterase-3 inhibitors, antibacterial activity, especially against methicillin-resistant *S. aureus*, antiepileptic, anticonvulsant agent	[37-40]
Furan	C_4_H_4_O	Has anticancer potential in three human cancer cell lines, such as breast cancer cells (MCF-7), lung cancer cells (A549), and melanoma cancer cells (A-375). Has good antibacterial activity on *Streptococcus pyogenes, Proteus vulgaris*, and *Escherichia coli*. Has antidiabetic activity	[41-43]
Alkanes	C_n_H_2n+2_ (straight and branched chain alkanes) C_n_H_2n_ (cyclic alkanes)	Anticancer activity of pulmonary carcinoma cells (A549), and antibacterial and cytotoxic.	[44,45]

**Non-volatile constituents**			

**Class**	Molecular formula*	**Bioactivity and medical benefit**	**Reference**

Flavones (Family of flavonoids)	C_15_H_10_O_2_	Antioxidant activity and anti-tyrosinase activity	[46]
Flavonols (Family of flavonoids)	C_15_HO_3_R_9_	Wound healing and anti-inflammatory activity	[22]
Flavonoid or bioflavonoid	C_6_-C_3_-C_6_	Treat kidney disorders, hypertension, wounds, diarrhea, rheumatism, shortness of breath, colds and coughs, respiratory tract infections, stomach pain and treat urinary tract infections	[47]
Two new flavonoids	1) C_18_H_16_O_8_ 2) C_20_H_20_O_8_	Antiproliferative cancer cells, especially flavonoids compounds 1) 3, 3’, 4’ -Trihydroxy- 6, 7, 8 -trimethoxy flavone, versus compounds of 2) 3-Hydroxy-6,7,8,3’,4’- pentamethoxy flavone.	[48]
Flavonoids	C_6_-C_3_-C_6_	Inhibition of xanthine oxidase (XO) and enzymatically is able to produce anti-free radicals	[49]
Chalcone	C_15_H_12_O	Anticancer, antibacterial, activity, cardiovascular infections, and antiparasitic.	[50-54]

* Source: pubchem.ncbi.nlm.nih.gov/compound. (National Library of Medicine). NO=Nitric oxide

## Polyphenolic Compounds

The leaf extract of *B. balsamifera* was reported to contain 18 polyphenol compounds, including 17 flavonoids and one phenyl ethanone, after analysis using high-performance liquid chromatography (HPLC) [55]. In addition, the flavonoid contents of this plant demonstrated antityrosinase and anticancer activities [56,57].

## Fatty Acids

Sembung leaves contain several fatty acids, as previously reported by Pang [7], including (11Z)-11-hexadecenoic acid, trans-2-undercenoic acid, 9-hexadecenoic acid, capric acid, and palmitic acid [58,59].

## Terpenoid Compounds

The essential oil obtained from *B. balsamifera* leaves was reported to have the following terpenoids: 1,8-cineole (20.98%), borneol (11.99%), β-caryophyllene (10.38%), camphor (8.06%), 4-terpineol (6.49%), a-terpineol (5.91%), and caryophyllene oxide (5.35%). These compounds are widely used as fumigants or volatile agents to kill insect pests, nematodes, and other pests [60].

## Flavonoids

Several studies reported that Sembung is rich in flavonoids such as 3,4,5-trihydroxy-3,7 dimethoxyflavonones, 3,4,5-trihydroxy-7-ethoxyflavanone, and the new biflavonoid, 3,-O-7-biluteolin, isolated using a Soxhlet extraction method [61]. A total of 27 compounds were identified using ultra-HPLC, including 16 flavonoid aglucagons, five flavonoid glycosides, five chlorogenic acid analogs, and one coumarin [22].

## Progress on Pharmacological Studies of *B. balsamifera*

Various experimental studies both *in vitro* and *in vivo* have reported the pharmacological activities of *B. balsamifera*, such as antioxidant, cytotoxic, antimicrobial, antifungal, anti-inflammatory, and hypolipidemic. Table-2 [13,22,56,62-80] summarizes some studies on the pharmacological activity of *B. balsamifera*.

**Table-2:** Pharmacological activities of *Blumea balsamifera*.

Pharmacological action	Experimental model	Extract	Positive control	Tested dose and Concentration	Result	Reference
Antioxidant	The leaves of Sembung are boiled and brewed as an ingredient in the traditional Balinese Loloh drink. The dried leaves are extracted by brewing. Determination of antioxidant levels using the DPPH free radical scavenging assay method	Fresh leaves and dry leaves	-	-	Dried sembung leaves made by boiling method had high antioxidant content, namely, 5.55 ± 0.01 mg GAE/g sample	[13]
	Hydro ethanol extract of Sembung leaves induced in diabetic rats previously induced with STZ	Hydro ethanol extract	Diabetic rat (STZ)	300 and 600 mg/kg	Increased levels of GSH and CAT as a marker of antioxidant activity	[68]
Antimicrobial	Sembung leaf extract was evaluated for its antibacterial activity using the disc diffusion test method and agar microdilution method	Essential oil extract Hexane Dichloromethane	Penicillin Chloramphenicol Tetracycline Gentamycin	Hexane (384 mg/disc) Dichloromethane (384 mg/disc) Essential oil (384 mg/disc)	Essential oils had the strongest inhibitory power with MIC concentrations of 150 mg/mL against *B. cereus* and 1.2 mg/mL against *S. aureus*	[62]
	Sembung essential oil was evaluated *in vitro* for its effectiveness against *Haemophilus parasuis* by observing MIC and MBC	Essential oil	C-: Tween-80 C+: *H. parasuis* bacteria in TSB media	2 × MIC 1 × MIC 0.5 × MIC 0.25 × MIC 0.125 × MIC	MIC and MBC were 0.625 and 1.25 mg/mL, respectively. When the extract concentration increased, the bacterial inhibition curve was stronger and *H. parasuis* cells were damaged after 4 hours of administration of the extract	[104]
	Sembung leaf extract against acne-causing bacteria, *Propionibacterium acnes*	Ethanol extract	Clindamycin 125 ppm	5%; 10%; 15%; 20%; 25%; 50%; 75% (Concentration of Sembung leaf extract)	The concentration of 75% showed the highest inhibitory power against *P. acnes* with a diameter of 2.26 cm MIC was 5% concentration, which was 0.93 cm	[73]
Antifungal	Sembung leaf extract was evaluated for its antifungal activity using the disc diffusion test method and agar microdilution method	Essential oil extract Hexane Dichloromethane	Penicillin Chloramphenicol Tetracycline Gentamycin	Hexane (384 mg/disc) Dichloromethane (384 mg/disc) Essential Oil (384 mg/disc)	Essential oils had antifungal properties against *C. albicans* and had the potential to be developed as a treatment and prevention of infectious diseases	[62]
	Sembung leaf extract was tested for its ability against several pathogenic fungi.	Ethyl acetate	Clotrimazole	30 mg	Had antifungal activity against *A. niger*, *T. mentagrophytes*, and *C. albicans*	[63]
	Sembung leaf extract was tested for its ability against Fluconazole resistant *C. albicans*	Ethyl acetate	Ketoconazole 15 mg	5%; 10%; 15%; 20% the concentration of Sembung leaf extract	The ethyl acetate extract of Sembung leaves had no inhibitory activity against fluconazole resistant *C. albicans*	[74]
	Lozenges made from Sembung leaves as drops and mouthwash against *C. albicans* which causes aphthous stomatitis	Ethanol extract	-	Pulvis Gummi Arabicum (PGA 5%: Mannitol 17.5%); F2(PGA 5%: 15%); F3(PGA 10%: mannitol 10%); F4(PGA 15%: Mannitol 5%); F5(PGA 17.5%: Mannitol 2.5%)	The best tablet formulation was the F3 formula with mannitol and PGA levels with a tablet weight of 400 mg Sembung extract had an activity against *C. albicans* with an inhibition zone of 10 mm and the lozenges meet the required standard of tablet properties	[107]
Anti-inflammatory activity	Flavonoids isolated from the Sembung plant were used to improve wound healing in Sprague-Dawley rats	-	30% glycerol	High dose (2.52 g/kg) Medium dose (1.26 g/kg) Low dose (0.63 g/kg) total flavonoids from *B. balsamifera*	The CD68 level was used as an anti-inflammatory marker that was elevated in the total flavonoid group Flavonoid glycosides had anti-inflammatory and wound healing activity	[22]
	*B. balsamifera* oil (BBO) obtained from sembung was used as an ingredient for burns healing in rats as an animal model	-	-	BBO was given to mice for 21 days and the rate of healing, decreased scabbing time, and re-epithelialization time were observed every 3 days for 21 days	BBO was able to reduce tissue water content, accelerate scab reduction time, and accelerate healing Growth factor expression occurred, but plasma inflammatory factor levels decreased	[76]
	*B. balsamifera* essential oil (BBEOs) was applied to determine its anti-inflammatory properties	-	-	BBEOs determined their chemical composition and anti-inflammatory activity using a model of skin injury in rat test animals induced by ultraviolet radiation (UV-B)	The application of BBEOs could effectively inhibit skin injury due to UV-B exposure by reducing the expression of inflammatory factors such as TNF-α, IL-6, and IL-10	[77]
	Nerve anti-inflammatory agent was isolated in *B. balsamifera* and tested on LPS-induced mic	-	-	The anti-inflammatory potential of *B. balsamifera* nerves was tested on LPS-induced mice and its activity was examined by measuring the release of NO in microglial BV-2 cells of mice	All isolates were able to show anti-inflammatory activity by inhibiting LPS-induced NO production in BV-2 cells mice These results indicate the bioactive compounds isolated from *B. balsamifera* have the potential to be developed as neuro-inflammatory agents	[64]
Hypolipidemic activity	Sembung leaf extract was used as a therapeutic agent for male reproductive problems due to high-fat diets in adult male rats	Ethanol	Sterile distilled water	2 mg/mL Sembung leaf extract	Sembung leaf extract was able to improve the histological profile of the testes of rats and increase the diameter of the seminiferous tubules and the number of spermatogenic cells in mice that were induced by a high-fat diet Sembung extract had hypolipidemic activity	[65]
	Lansau or traditional ingredients of the Muna tribe, Southeast Sulawesi, Indonesia were used as hypolipidemic agents based on LDL parameters	Traditional herb or Lansau consisted of various spices and traditional plants, one of which is *B. balsamifera*	Simvastatin	250 mL and 500 mL for the Lansau dosage	Elderly infusions containing traditional plants including *B. balsamifer*a could be used as anti-hyperlipidemia based on decreased parameters of LDL levels in rat test animals	[78]
Anti-Infertility activity	Traditional Dayak plants including *B. balsamifera* were used as an anti-infertility ingredient in Swiss Webster (SW) mice	-	Sterile aquabidest	2.6 mg/kg bb	*B. balsamifer*a has the highest anti-infertility activity compared to other traditional plant extracts	[66]
Hepatotoxicity and hepatoprotective activity	Leaf extract of *B. balsamifera* was tested on male mice to determine hepatotoxicity	Ethanol extract	Na-CMC 1%	The dose were 2; 2.5; 3.2; 4 g/kg body weight intraperitoneally (IP)	The liver cells, cytoplasm, nucleus, and sinusoid in the liver of mice were damaged due to several changes in liver color and texture	[79]
	The leaf extract of *B. balsamifera* was tested for growth inhibition activity of human hepatocellular carcinoma cells (McA-RH777, and HepG2, respectively) in tested mice	Methanol extract	Induction of carcinoma cells without extracting	-	The anti-proliferation effect increased significantly and was able to reduce ligands related to tumor cell proliferation These findings provide a report that Sembung has hepatoprotective potential	[67]
Anti-diabetic activity	*B. balsamifera* (HEBB) leaf extract was evaluated for antidiabetic activity in STZ-induced rats	Hydro-ethanol extract	STZ and Glibenclamide (5 mg/kg/bw, po)	HEBB 300 and 600 mg/kg	Significant changes in serum lipid profiles and enzymes marker	[68]
Gastro-protective activity	Combination of hot water extract *Glycyrrhiza glabra, Alyxia reinwardtii, B. balsamifera* to determine gastro-ptotective activity in aspirin-induced mice	Hot water extract	Aspirin 450 mg/kg BW and Sucralfate 360 mg/kg BW	*G. glabra* 273 mg/kg BW; Sembung leaf 457.5 mg/kg BW; and *A. reinwardtii* with various doses, namely 100 mg/kg BW (K1); 200 mg/kg (K2); 300 mg/kg BW (K3)	The combination of herbal extracts was able to significantly provide a protective effect indicated by the small or small area of the peptic ulcer	[69]
Antitumor activity	*B. balsamifera* essential oil extract was tested for its antitumor activity	Essential oil extract	Trolox	IC_50_ 0.6342 mL/mL LC_50_ 65 mg/mL	Has antitumor activity tested on shrimp larvae	[70]
Anticancer activity	Fractionation of *B. balsamifera* leaf ethyl acetate extract to be tested for the anticancer ability of KB, MCF-7, and NCI-H187	Ethyl acetate extract fraction	-	-	Compounds 2,4, and 9 were active against KB cells with IC50 values 17.09, 47.72, and 17.83 mg/mL Compounds 2,3, and 5 showed moderate activity against NCI-H187 cells with IC50 values of 16.29, 29.97, and 20.59 mg/mL Leteolin-7-methyl ether (9) had strong cytotoxicity against human lung cancer cells (NCI-H187) with an IC50 of 1.29 mg/mL and moderate toxicity to oral cancer cells (KB) with an IC50 of 17.83 mg/mL	[56]
Immunomodulator for SARS-CoV-2	Genes that can be involved in the regulation of biosynthesis of active compounds from *B. balsamifera*	-	-	-	The CCL and FPS genes had an immunostimulatory induction role, protect the hepatocytes from lipid peroxidation and catalytic activity	[71,80]

MIC=Minimum inhibition concentration, *B. cereus=Bacillus cereus*, *S. aureus=Staphylococcus aureus*, DPPH: 2,2-diphenylpicrylhydrazyl, *A. niger=Aspergillus niger*, *T. mentagrophytes=Trichophyton mentagrophytes*, *C. albicans=Candida albicans*, NO=Nitric oxide, LPS=Lipopolysaccharide, STZ=Streptozotocin, GSH=Glutathione, CAT=Catalase

## Antioxidant Activity

*B. balsamifera* extract has demonstrated high antioxidant activity. Fresh and dry leaf extracts from *B. balsamifera* have been used as a traditional drink by Balinese people in Indonesia called Loloh. It was reported that dried leaves of *B. balsamifera*, obtained by brewing, had a tannin content of 13.15±0.11 mg GAE/g, while boiled dried leaves showed a high antioxidant capacity of 5.55±0.01 mg GAE/g [13]. Apart from *B. balsamifera*, other species such as *Blumea lanceolaria* were also reported to have good health benefits. The antioxidant activity of the methanol extract of *B. lanceolaria* leaves was assessed through three different methods 2,2-diphenylpicrylhydrazyl, ferric reducing antioxidant power test, and total phenolic content test, showing an antioxidant activity of 302.37±59.78 mg/100 g, 4.60±0.17 mg/100 g, and 1298.93 mg/100 g [81], respectively.

Essential oils obtained from *B. balsamifera* have been widely used in various countries, especially in tropical Asia. Parts of the *B. balsamifera* plant were also reported to contain different essential oils. The highest yield of essential oil, 0.65 mL/100 g, was obtained from young leaves. In addition, shoots and young leaves also showed the strongest antioxidant activity. Dimethoxydurene, β-caryophyllene, and a-caryophyllene, which played an important role in the plant’s antioxidant activity, have the potential to be developed as ingredients in the cosmetic and medicinal industries [58].

## Antimicrobial Activity

*B. balsamifera* leaves have been used in the treatment of bacterial infections. The essential oil, and *n*-hexane, dichloromethane, and methanol extracts of *B. balsamifera* have been evaluated for their antibacterial activity using agar diffusion and microdilution methods. The essential oil gave the best results with a minimum inhibition concentration (MIC) value of 150 mg/mL against *Bacillus cereus* and a MIC of 1.2 mg/mL against *Staphylococcus aureus*. Besides, the *n*-hexane extract also showed good bactericidal activity [62]. Compounds with high antioxidant activity, such as d-elements, a-cubenene, caryophyllene, caryophyllene epoxide, g-eudesmol, xanthoxylin, and a-eudesmol, were identified from the essential oil of *B. balsamifera* [82].

The ethanol extract of the stems, roots, and leaves of *B. lanceolaria* also showed good antimicrobial activity against *S. aureus*, namely, 10-12 mm, when compared with standard antibiotics, 2-10 mm [23]. *Blumea lacera* leaf extract, for example, was reported to have antimicrobial activity [83].

## Antifungal Activity

Antifungal activity had also been reported in *B. balsamifera* extract. Antifungal activity tests have been carried out on *Aspergillus niger*, *Trichophyton mentagrophytes*, and *Candida albicans*. Compounds with antifungal activity in Sembung leaves include icthyothereol acetate and cryptomeridiol [63]. The antifungal role of *B. lacera* was studied against *Aspergillus flavus*, *A. niger*, *Alternaria* sp., *Penicillium* sp., and *Fusarium* sp. Almost all extracts from both methanol, acetone, and water extracts were reported to have antifungal activity, when compared to standard carbendazim [84].

## Anti-inflammatory Activity

Scientific evidence has demonstrated *B. balsamifera* anti-inflammatory activity. When the wound healing process occurs, it will go through several stages such as inflammatory response, migration, proliferation, and regeneration of new tissue [85]. Total flavonoids isolated from *B. balsamifera* were used as a skin wound healing agent in Sprague-Dawley rats. The healing activity was determined by measuring CD68 levels, vascular endothelial growth factor, transformation growth factor-β1, and hydroxyproline. The results of that study indicated that flavonoids were key in the successful wound healing process, increasing the expression of growth factors [22].

The study of the anti-inflammatory process was also carried out using *Blumea aurita*. *B. aurita* had the highest percentage of inhibition of edema (EI% = 53%) after 4 h of oral administration of the extract at a dose of 400 mg/kg, followed by 6 h (EI% = 67%) at a dose of 800 mg/kg, with albino Wistar mice [86]. The aqueous extract of *Blumea mollis* was also used in an acute and chronic anti-inflammatory therapy on carrageenan-induced rat leg edema [87].

In addition, *B. balsamifera* extract was also reported to have anti-inflammatory neuroprotective activity by reducing nitric oxide in lipopolysaccharide-induced rat microglial BV-2 cells. This inhibition may have occurred due to the interaction of the bioactive compounds of *B. balsamifera* extract with iNOS protein [64].

## Hypolipidemic Activity

Hypolipidemic drug agents lower lipoprotein concentrations, transporting excess cholesterol, and triglycerides in the blood [88]. The antihyperlipidemic nature or potential in plants is important in reducing atherosclerosis [89]. The Sembung plant has been used as an antihyperlipidemic agent *in vitro* in 3-4 months old adult male Wistar rats (*Rattus norvegicus*) with induced high-fat feed. The Sembung extract had anti-hyperlipidemic activity by increasing spermatocytes [65]. A traditional drink from Southeast Sulawesi, Indonesia, “Lansau,” made from 44 traditional ingredients (including Sembung) showed that Lansau’s ethanol extract was antihyperlipidemic, reducing fat degeneration and being able to repair cell damage at a dose of 27,628 mg/kg [90].

## Anti-infertility Activity

Infertility can be defined as a medical condition that can cause psychological and physical harm. Several factors such as age, tubal factors in women, obesity, significant reduction in semen parameters, cigarette consumption, and excessive alcohol consumption in men could trigger infertility [91]. Ethnopharmacological surveys on uses of traditional medicines against infertility have been widely completed around the world [92].

Local plant extracts from the Dayak tribe, Kalimantan, Indonesia, such as *B. balsamifera*¸ *Croton tiglium*, *Metroxylon sagu*, and *Fagraea racemosa* Jack, have been used as anti-infertility substances *in vitro*. All extracts were able to inhibit the estrous and metestrus cycles. A decrease in the corpus luteum and fetus was attributed to the anti-infertility effect of the extract against the inhibition of folliculogenesis. Researchers also reported that *B. balsamifera* extract had promising anti-infertility activity compared to other plant extracts used in this study [66].

## Hepatoprotective Activity

The incidence of liver diseases affects millions of people worldwide. The prevalence of liver cirrhosis from autopsy studies ranges globally from 4.5% to 9.5% of the global population [93,94], with more than 50 million people worldwide (adult population) potentially affected by chronic liver disease [95].

A phytotherapy approach in the development of modern medicines is still very much needed [96]. The benefits of the phytochemical compounds in *B. balsamifera* have been widely used to improve physiological disorders and other degenerative diseases. Researchers also mentioned that the methanol extract of *B. balsamifera* (BME) induced growth and developmental inhibition of human hepatocellular carcinoma cells (McA-RH7777 and HepG2, respectively) in mice. These results were confirmed by the antiproliferative effect of BME, which increased slightly but significantly reduced the level of proliferation-related ligand (APRIL) stimulating tumor cells [67].

The hepatoprotective effect was also reported for the ethanol extract of *B. lacera*, which showed minimal damage to liver structures, decreased aspartate aminotransferase, Alkaline phosphatase, and bilirubin in ethanol-induced rats [97].

## Antidiabetic Activity

The use of *B. balsamifera* as an antidiabetic in traditional medicine has been widely used, especially in Ayurvedic medicine in India. The administration of a hydro-ethanolic extract from *B. balsamifera* (HEBB) at doses of 300 and 600 mg/kg, in streptozotocin (STZ)-induced diabetic rats, showed a decrease in blood glucose, lipid profile, serum marker enzymes, and levels of glutathione and catalase, when compared with the diabetes control group [68].

Other species such as *B. lacera* D.C (Asteraceae) was also reported to have antidiabetes benefits. Methanol extract *B. lanceolaria* (MEBL) and water extract *B. lanceolaria* were tested on hyperglycemic rats induced by STZ. Treatment with MEBL at doses of 200 and 400 mg/kg BW was able to lower blood glucose levels, increasing glycated hemoglobin, restoring lipid levels, rejuvenating pancreatic beta cells, and increasing the level of insulin secretion in the blood [98].

## Gastroprotective Activity

Digestive disorders such as peptic ulcers are caused by an imbalance of aggressive factors (gastric acid and pepsin) and defense factors (mucosal secretion, bicarbonate secretion, and to mucosal epithelial regeneration) [69]. Some traditional plants, such as Sembung can be used as a medicinal ingredient for gastrointestinal disorders. Experimentally, the gastroprotective effect of herbal ingredients was applied to mice in a model of aspirin-induced gastric ulcers. This study confirmed that the herbal extract formulation using Sembung was able to provide a gastroprotective effect, with the lowest eosinophil and mast cells count [69,99].

## Antitumor Activity

The essential oil extract of *B. balsamifera* was reported to have 42 types of chemical components, which were tested for their antitumor activity using the method of shrimp-larvae mortality determination, which is a simple, convenient, and inexpensive method of determining internal biological activity initiated by Jiang *et al*. [70]. This method has been widely used in determining the toxic components and contaminants in food, as well as the determination of biological activity [100]. The method was carried out using 25-30 larvae in each group. Treatment was given when shrimp eggs hatched (1% DMSO was added to dissolve the sample). The survival rate was observed for 24 h at room temperature. The treatment at 65 mg mL showed strong cytotoxicity to shrimp larvae. In conclusion, this extract was set as potential for further studies on antitumor activity [70].

## Anticancer Activity

Cancer is a potentially life-threatening disease with more than 100 different types already occurring due to molecular changes in cells [101]. Cancer is also reported to be the third leading cause of death worldwide after cardiovascular and infectious diseases [102]. Medicinal plants have been reported as potential cancer treatment agents, as about 50% of compounds derived from medicinal plants demonstrate anticancer activity [103].

A study reported the anticancer activity of the BME on mice induced by human hepatocellular carcinoma cells (McA-RH7777 and HepG2, respectively). BME was able to reduce the level of APRIL, which is able to stimulate tumor cells. APRIL is a new member of the tumor necrosis factor, which is reported to stimulate tumor cell growth, modulate tumor cell apoptosis, and regulate humoral immunity [104,105].

The anticancer potential of other species such as *Blumea eriantha* was also tested *in vitro* using the microtetrazolium assay (MTT) test on cervical cancer cell (HeLa) and B16F10 cell gallus. The expressions of the p53 and Bcl-2 genes associated with apoptosis were also determined. The results showed that the methanol extract of *B. eriantha* induced strong antioxidant and anticancer properties. In addition, this extract was also to prevent cancer cell metastasis [106].

## Immunomodulator Activity for Severe Acute Respiratory Syndrome-Coronavirus (SARS-CoV-2) in CoV Disease (COVID)-19

Novel COVID-2019, related with SARS, has been declared as a global pandemic causing deaths in 216 countries worldwide [107]. Until now, no vaccine or special treatment for SARS-CoV-2 has been available despite the extraordinary recent research efforts [108]. The use of drugs such as remdesivir, lopinavir/ritonavir, and hydroxychloroquine has been widely claimed to reduce the symptoms of COVID-19. To date, 200 clinical trials have been registered on the clinicaltrials.gov site. However, the expediency of drugs being studied is still unclear [109].

Therefore, the use of traditional medicinal plants is recommended by some for management or increasing the immune status of patients with COVID-19. A report showed that 90% of traditional medicines resulted in 90% recovery in 214 patients treated for COVID-19 [110]. The use of traditional medicine also claimed to be able to prevent COVID-19 infection in healthy people and to increase the immunity of patients with mild or severe COVID-19 symptoms [110]. In Indonesia, the use of “Empon-empon” (In Java) or Loloh (Balinese) made from traditional medicinal plants is also believed to have a role in modulating the immune system in COVID-19 patients [71].

**Figure-2:**
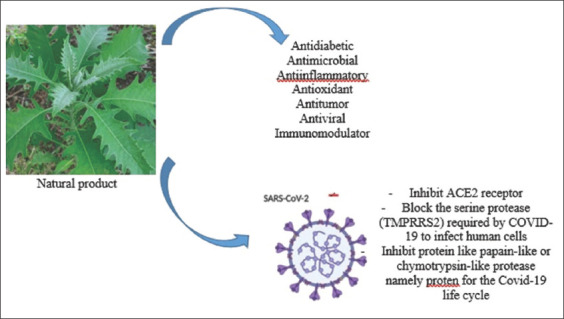
Summary of the potential of natural products from the Sembung plant (*Blumea balsamifera*).

Therapeutic agents against COVID-19 can be divided into several categories based on specific pathways: (1) Preventing the synthesis and replication of viral RNA, (2) blocking the virus in order not to bind to human cell receptors or by inhibiting the assembly process of viral genetic material, (3) restoring the innate immunity of the host, and (4) acting on specific receptors or enzymes so that the virus does not enter the host cell. Until now, there is still no specific scientific data mentioning *B. balsamifera* as an antiviral agent for COVID-19. Therefore, we suggest that the antiviral potential of its phytocomponents could be tested through computational studies (*in silico* studies), *in vitro*, and *in vivo*. Figure-2 summarizes the potency of *B. balsamifera* as a medicinal agent and possible anti-SARS-CoV-2.

## Future Prospective

More exploration and research have been carried out to verify the benefits of *B. balsamifera*, which is widely used against various diseases by people in several countries. The diversity of its constituents, molecular structure, bioactivity, and pharmacological studies has been extensively described in this review. Researchers have also demonstrated the efficacy of this plant in treating diseases (Table-2). However, further and more detailed studies should be carried out to assess the use of this plant in a number of other experimental animals such as Ferret (*Mustela putorius*) [111], or Zebrafish (*Danio rerio*) as well as on human subjects (can be considered) [112]. It has been reported that the leaves of *B. balsamifera* are the most widely used part in extracts (water, methanol, and ethanol) in studies against cancer, diabetes, hyperlipidemia, infertility, and infectious diseases. Its biological activity is still not fully proven, so other clinical studies would be needed [3].

Some other species of this family have been the focus of some research. *B. lacera* was reported to be antidiabetic, antifungal, and hepatoprotective; *B. eriantha* was able to demonstrate its anticancer activity. Therefore, research should focus in the identification and isolation of bioactive compounds according to the known pharmacological activity of *B. balsamifera*. Especially during the COVID-19 pandemic, authentic Indonesian herbal ingredients are needed to assess their benefits in modulating the immune system.

## Conclusion

The Sembung plant (*B. balsamifera*) has proven to possess important biological activities with additional potential to be developed as a candidate for future essay-based isolation and identification of its bioactive.

## Authors’ Contributions

IGW designed and prepared the manuscript. IMJ made a critical comment in this manuscript. Both authors read and approved the final manuscript.
